# Association of Primary Care Continuity With Outcomes Following Transition to Adult Care for Adolescents With Severe Mental Illness

**DOI:** 10.1001/jamanetworkopen.2019.8415

**Published:** 2019-08-02

**Authors:** Alène Toulany, Thérèse A. Stukel, Paul Kurdyak, Longdi Fu, Astrid Guttmann

**Affiliations:** 1 Division of Adolescent Medicine, Hospital for Sick Children, Toronto, Ontario, Canada; 2 ICES, Toronto, Ontario, Canada; 3 Department of Pediatrics, University of Toronto, Toronto, Ontario, Canada; 4 Institute for Health Policy Management and Evaluation, University of Toronto, Toronto, Ontario, Canada; 5 Department of Psychiatry, Faculty of Medicine, University of Toronto, Toronto, Ontario, Canada; 6 Institute for Mental Health Policy Research, Centre for Addiction and Mental Health, Toronto, Ontario, Canada; 7 Division of Paediatric Medicine, Hospital for Sick Children, Toronto, Ontario, Canada

## Abstract

**Importance:**

Adolescents with severe mental illness often age out of pediatric care without a clear transfer of care to adult services. The extent to which primary care provides stability during this vulnerable transition period is not known.

**Objective:**

To analyze the association between primary care continuity during the transition from pediatric to adult care and need for acute mental health services in young adulthood.

**Design, Setting, and Participants:**

This population-based cohort study used linked health and demographic administrative data for all adolescents aged 12 to 16 years with severe mental illness ascertained by hospitalization for schizophrenia, eating disorder, or mood disorder between April 1, 2002, and April 1, 2014, in Ontario, Canada. Participants were followed up through March 31, 2017. Data were analyzed from July 2018 to January 2019.

**Exposures:**

Continuous primary care (same physician as baseline [age 12-16 years] always or sometimes), discontinuous primary care (visits to a primary care physician during the transition period who was not the patient’s usual physician), and no primary care during the transition period (age 17-18 years).

**Main Outcomes and Measures:**

Mental health–related hospitalizations and emergency department visits in young adulthood (age 19-26 years) adjusted for sex, rurality, neighborhood income, mental illness type, and health service use before transition.

**Results:**

Among 8409 adolescents with severe mental illness (5720 [68.0%] female; mean [SD] age, 14.8 [1.2] years), 5478 (65.1%) had continuous primary care, 2391 (28.4%) had discontinuous primary care, and 540 (6.4%) had no primary care during the transition period. Youths with no primary care during transition were more likely to be male (57.2%), have lower socioeconomic status (31.5%), and have no usual primary care practitioner at baseline (25.6%). Compared with continuous care, patients with discontinuous and no primary care had an increased rate of mental health–related hospitalization in young adulthood (adjusted relative rate, 1.20; 95% CI, 1.10-1.30; and adjusted relative rate, 1.30; 95% CI, 1.08-1.56, respectively).

**Conclusions and Relevance:**

In the context of decreasing outpatient specialist mental health visit rates following transition to adult care, ensuring adequate access to primary care during this vulnerable period may improve mental health outcomes in young adulthood.

## Key Points

**Question:**

Is primary care continuity during the transition from pediatric to adult care services associated with better outcomes in young adulthood for adolescents with severe mental illness?

**Findings:**

In this population-based cohort study of 8409 adolescents aged 12 to 16 years with severe mental illness, there was a 30% increase in risk of mental health–related hospital admission in young adulthood (age 19-26 years) for those with no primary care physician and a 20% increase for those with discontinuous primary care during transition to adult care compared with continuous care.

**Meaning:**

For adolescents with severe mental illness, continuity with a primary care physician during transition to adult care was associated with decreased mental health–related hospitalizations and emergency department visits.

## Teaser

This population-based cohort study assesses the association between primary care continuity from pediatric to adult health care services during adolescence and outcomes in young adulthood for patients with severe mental illness in Ontario, Canada.

## Introduction

Mental disorders are a critical health issue affecting 1 in 5 adolescents and young adults at any given time.^1,2^ Most mental disorders begin in adolescence and early adulthood and are major contributors to the burden of disease in young people.^3,4,5,6^ Most adolescents with severe mental disorders require a transition from pediatric to adult care practitioners because of the chronicity and functional impact of these disorders. In the United States, most youth transition to adult care between the ages of 18 and 21 years. In Ontario, Canada, government funding models mandate that the transfer of all pediatric care occur by age 18 years. This transition also includes mental health care, as most child psychiatrists practice in pediatric hospitals. However, under exceptional circumstances, the Ontario government will pay pediatrician billing claims for adult patients aged 18 to 22 years for ongoing management of chronic conditions. While most children and youth in Ontario have their primary care delivered by family physicians, a substantial number of pediatricians also provide primary care to children, predominantly in larger urban centers.^7^ Children with complex health care needs and those from families with higher income are more likely to have a pediatrician as their primary care practitioner.^7^ When primary care is delivered by pediatricians, the mandatory transfer of care to a family physician at age 18 may be in addition to the transition of mental health care for adolescents with severe mental illnesses.

Poor health outcomes following transition have been described for several populations such as those with sickle cell disease,^8^ congenital heart disease,^9^ diabetes,^10^ and organ transplantation.^11,12^ Similarly, large declines in mental health service use have been described after the transition from youth to adult services for youth with mental illness.^13,14^ This decrease is likely due to pediatric and adult mental health systems generally functioning as separate entities with marked differences in approach, eligibility, and care philosophies.^15,16,17,18^ Internationally, researchers are demonstrating significant gaps in systems of care between pediatric and adult mental health services.^14,17,19,20,21,22,23,24^ The first systematic attempt to study the policy, process, outcome, and experience of transition for youth with mental illness demonstrated that almost half of youth “fell through the care gap”^14^ between child and adult systems, and those who successfully transitioned received care that was poorly planned, executed, and experienced.

Evidence to support whether continuity of care is associated with improved health outcomes in transition-aged youth with chronic medical or mental illness is sparse,^10,25^ although improving continuity has been identified as a priority for policy makers and professional organizations.^26,27^ Primary care practitioners are described as a critical component of transition planning; however, there is little evidence testing models for how they could or should be incorporated into the transition planning process for adolescents and young adults with mental illness.^28,29^ Leveraging the availability of linked administrative health data for the entire population of Ontario, our objective was to determine whether there is an association of primary care continuity during the transition period and poor mental health outcomes measured by the need for acute mental health care services in young adulthood. We hypothesized that across patients with a diverse group of severe mental health conditions, continuous primary care during transition will be associated with a decrease in mental health–related hospitalizations and emergency department visits.

## Methods

### Data Sources and Study Cohort

This was a population-based cohort study of transition-aged youth (age 12-26 years) using administrative health data available at ICES (formerly the Institute for Clinical Evaluative Sciences), an independent, nonprofit organization that evaluates health care services in the Province of Ontario, Canada (population approximately 14 million). Ontario provides universal health care to its residents, including ambulatory and in-hospital medical and mental health care. The cohort (dates of birth, April 1, 1990, to April 1, 1997) was ascertained between the ages of 12 and 16 years using hospitalization data from April 1, 2002, to April 1, 2014. We examined the pattern of primary health care across the transition period, from ages 17 to 18 years, and evaluated outcomes in the period following transition, from ages 19 to 26 years. Patients were followed up until their 27th birthday or the end of the study period (March 31, 2017), corresponding to a minimum follow-up period of 1 year and maximum of 8 years. Data analysis was performed from July 2018 to January 2019.

At ICES, patient-level records across several databases are linked using unique encoded identifiers. The Canadian Institute for Health Information Discharge Abstract Database and Ontario Mental Health Reporting System were used to identify the index mental health hospitalization, define the adolescent cohort, and ascertain outcome events. A list of all data sources is provided in eTable 1 in the Supplement.

The cohort included all adolescents with a history of hospitalization longer than 72 hours between the ages of 12 and 16 years for a severe mental illness, including schizophrenia and related psychotic disorders (SZ), eating disorders (ED), and mood disorders (MD). Conditions were sorted hierarchically into 3 mutually exclusive condition categories beginning with SZ, followed by ED, then MD. The *International Classification of Diseases, Tenth Revision* (*ICD-10*) and *Diagnostic and Statistical Manual of Mental Disorders* (Fourth Edition) (*DSM-IV*) diagnostic codes for SZ, ED, and MD were used to construct the cohorts. Patients were excluded from the study if they died before age 19 years, did not have continuous residency in Ontario between ages 12 to 19 years, or received primary care in a community health center (physicians do not bill the government for services provided at these organizations).

As a prescribed entity under Ontario’s Personal Health Information Protection Act, ICES has the authority to enable health system research without individual patient consent under a strict privacy and security framework. Approval to complete this study was granted by the University of Toronto and Hospital for Sick Children research ethics boards. This study followed the Strengthening the Reporting of Observational Studies in Epidemiology (STROBE) reporting guideline for cohort studies.

### Exposure Categories

Patients were divided into 3 exposure categories based on whether they received care from the same (continuous care), different (discontinuous care), or no primary care physician during the transition period to adult care (ages 17-18 years). The usual primary care practitioner (either family physician or pediatrician) was assigned at age 17 years by using enrollment data from the Client Agency Program Enrollment database covering the majority of Ontario family practices, or Ontario Health Insurance Plan physician primary care visit billings to assign those seeing other family physicians or pediatricians. We defined continuous primary care as visits during the transition period (ages 17-18 years) to the usual primary care practitioner always or sometimes. Discontinuous primary care was defined as visits to a primary care physician during the transition period who was not the patient’s usual physician.

### Outcomes

The primary outcome was rate of mental health–related hospitalizations measured per person-years after transition to adult care services from ages 19 to 26 years. We followed adolescents to March 31, 2017, corresponding to a minimum follow-up period of 1 year and maximum of 8 years. Mental health–related hospitalizations were defined as any mental health code (*ICD-10* F00-F99) as the most responsible diagnosis in hospital databases (eTable 2 in the Supplement). Our secondary outcomes included mental health–related emergency department visits, defined as any mental health code (*ICD-10* F10-F99) in the main diagnostic field or a self-harm code (*ICD-10* X60-X84) in any diagnostic field, and all-cause mortality after transition to adult care services (eTable 2 in the Supplement).

### Covariates

Patient-level demographic, clinical, and health service use covariates were included in the analysis as potential confounders. Demographic covariates included sex, rural residence, and neighborhood income quintile. Rural residence was defined according to Statistics Canada’s classification of rural and small town areas (population <10 000). Neighborhood income quintile was used as a measure of socioeconomic status. The patient’s type of mental illness and comorbidities were considered possible confounders as the trajectory and potential for resolution of symptoms differs by condition. In our final models, we adjusted for sociodemographic factors and mental illness comorbidities using the categories: SZ alone, SZ and MD, ED alone, ED and MD, and MD alone. We controlled for severity of illness in 2 ways. We used mental health–related hospitalizations and emergency department visits before (ages 12-16 years) and during (ages 17-18 years) the transition period as a measure of more severe illness. Similarly, we controlled for mental health visits by specialty of practitioner during transition as a proxy for both disease activity and severity of illness, assuming those who were sicker were more likely to see a psychiatrist for their outpatient mental health care.

### Statistical Analysis

We compared descriptive patterns of health service use, overall and by mental illness type, before (ages 12-16 years), during (ages 17-18 years), and after (ages 19-26 years) the transition period to adult care. Owing to the unequal transition periods and variable length of follow-up among participants, we calculated annualized rates of outcomes for each period before, during, and after transition.

We used multivariate Poisson regression to examine the association between continuity of primary care during transition and outcomes after transition accounting for variable length of follow-up with an offset parameter and using robust standard errors to correct for potential variance misspecification.

Variables were selected a priori based on clinical significance or evidence from the literature. We sequentially added blocks of variables into the models to examine their effects on confounding beginning with sex, rural residence and income, type of mental illness, mental health admissions before transition, mental health admissions during transition, and mental health visits by specialty during transition (eTable 3 in the Supplement). There were no prespecified hypotheses regarding interactions. All variables were retained in the final model regardless of statistical significance. The associations were expressed as adjusted relative rates (aRRs) with 95% confidence intervals. A subgroup analysis was also performed including only those youths with a family physician as their usual primary care practitioner at baseline (eTable 4 and eTable 5 in the Supplement).

Analyses were conducted using SAS statistical software version 9.4 (SAS Institute Inc). All tests were 2-tailed and *P* < .05 was considered statistically significant.

## Results

### Cohort Demographic and Clinical Characteristics

A total of 9170 adolescents aged 12 to 16 years had a history of hospitalization greater than 72 hours duration for SZ, ED, or MD from April 1, 2002, to April 1, 2014. We excluded 761 adolescents, including those who died before age 19 years (n = 41), had noncontinuous Ontario residency in the baseline period (n = 249), or received primary care in a community health center (n = 471). The final cohort included 8409 youths (5720 [68.0%] female; mean [SD] age, 14.8 [1.2] years) with severe mental illness. Of these participants, 5478 (65.1%) had continuous primary care, 2391 (28.4%) had discontinuous primary care, and 540 (6.4%) had no primary care during the transition period. Youths with no primary care practitioner during transition were more likely to be male (57.2%), have lower socioeconomic status (31.5%), live in a rural area (21.0%), and have no usual primary care practitioner at baseline (25.6%) (Table 1). They were slightly more likely to have a diagnosis of SZ and less likely to have a diagnosis of ED. They were similar in terms of number of mental health–related admissions and emergency department visits before transition (ages 12-16 years) but less likely to have an admission or emergency department visit during transition (ages 17-18 years).

**Table 1.** Demographic Characteristics and Health Service Use Before (Ages 12-16 Years) and During (Ages 17-18 Years) Transition by Pattern of Primary Care During Transition for 8409 Participants

Variable	Pattern of Primary Care During Transition, No. (%)		
	Discontinuous (n = 2391)	None (n = 540)	Continuous (n = 5478)
Female	1568 (65.6)	231 (42.8)	3921 (71.6)
Age at index hospitalization, mean (SD), y	14.7 (1.2)	14.5 (1.3)	14.9 (1.2)
Neighborhood income quintile			
1 (Lowest)	532 (22.2)	170 (31.5)	932 (17.0)
2	451 (18.9)	112 (20.7)	1011 (18.5)
3	439 (18.4)	90 (16.7)	1115 (20.4)
4	459 (19.2)	81 (15.0)	1213 (22.1)
5 (Highest)	510 (21.3)	87 (16.1)	1207 (22.0)
Rurality			
Urban	2103 (88.0)	427 (79.0)	4883 (89.1)
Usual primary care practitioner before transition			
Family physician	1553 (65.0)	354 (65.6)	5393 (98.4)
Pediatrician	639 (26.7)	48 (8.9)	85 (1.6)
None	199 (8.3)	138 (25.6)	NA
Type of mental illness			
Schizophrenia^a^	267 (11.2)	86 (15.9)	451 (8.2)
Eating disorder	417 (17.4)	53 (9.8)	933 (17.0)
Mood disorder	1707 (71.4)	401 (74.3)	4094 (74.7)
Mental health–related emergency department visits before transition, No.			
0	1416 (59.2)	344 (63.7)	3313 (60.5)
1	594 (24.8)	138 (25.6)	1455 (26.6)
2	241 (10.1)	42 (7.8)	479 (8.7)
≥3	140 (5.9)	16 (3.0)	231 (4.2)
Mental health–related admissions before transition, No.			
0	1247 (52.2)	301 (55.7)	3008 (54.9)
1	574 (24.0)	140 (25.9)	1375 (25.1)
2	266 (11.1)	58 (10.7)	543 (9.9)
≥3	304 (12.7)	41 (7.6)	552 (10.1)
Mental health–related emergency department visits during transition, No.			
0	1888 (79.0)	460 (85.2)	4385 (80.0)
1	368 (15.4)	64 (11.9)	789 (14.4)
2	99 (4.1)	15 (2.8)	210 (3.8)
≥3	36 (1.5)	<6	94 (1.7)
Mental health–related admissions during transition, No.			
0	1868 (78.1)	468 (86.7)	4352 (79.4)
1	289 (12.1)	45 (8.3)	637 (11.6)
2	100 (4.2)	10 (1.9)	239 (4.3)
≥3	134 (5.6)	17 (3.1)	250 (4.6)
Mental health–related visits by specialty during transition			
Psychiatrist (any)	1219 (51.0)	138 (25.6)	2800 (51.1)
Family physician and/or pediatrician	518 (21.7)	11 (2.0)	1659 (30.3)
No mental health visits	654 (27.4)	391 (72.4)	1019 (18.6)

Abbreviation: NA, not applicable.

^a^ Includes schizophrenia, delusional, and nonorganic psychotic disorder.

### Primary Care During Transition

Family physicians were the usual primary care practitioners at baseline for 7300 adolescents (86.8%). Among those with a family physician at baseline, 5393 (73.9%) continued to see the same physician during transition, whereas 1553 (21.3%) saw a different one and 354 (4.8%) had no primary care (Table 2). Adolescents with SZ, however, had the least continuity during transition with 185 (27.0%) receiving care from a different physician and 58 (8.5%) having no primary care. Overall, transition to a family physician was successfully achieved by 639 adolescents (82.8%) with a pediatrician as their usual primary care practitioner. In addition, 199 adolescents (59.1%) with no usual primary care practitioner at baseline successfully transitioned to a family physician.

**Table 2.** Pattern of Primary Care Across the Transition Period (Ages 17-18 Years) by Type of Mental Illness for 8409 Participants

Usual Primary Care Practitioner (12-16 y)	Primary Care During Transition (17-18 y)	No. (%)		
		Schizophrenia (n = 804)^a^	Eating Disorder (n = 1403)	Mood Disorder (n = 6202)
Family physician	No primary care	58 (8.5)	35 (3.0)	261 (4.8)
	Continuous primary care	441 (64.5)	916 (78.1)	4036 (74.1)
	Discontinuous primary care	185 (27.0)	222 (18.9)	1146 (21.1)
	Total, No.	684	1173	5443
Pediatrician	No primary care	13 (16.1)	8 (4.1)	27 (5.4)
	Continuous primary care	10 (12.3)	17 (8.7)	58 (11.7)
	Discontinuous primary care (family physician)	58 (71.6)	170 (87.2)	411 (82.9)
	Total, No.	81	195	496
None	No primary care	15 (38.5)	10 (28.6)	113 (43.0)
	(Discontinuous) primary care	24 (61.5)	25 (71.4)	150 (57.0)
	Total, No.	39	35	263

^a^ Includes schizophrenia, delusional, and nonorganic psychotic disorder.

The mean (SD) annualized rate of mental health–related primary care visits increased across transition for youth with SZ and MD (eTable 6 in the Supplement). Visits to psychiatrists increased during transition (ages 17-18 years) for all mental illness types but decreased after transition to rates comparable to baseline for SZ and significantly below baseline for ED and MD. During transition, mental health–related admissions and emergency department visits increased for youths with SZ but were stable for those with ED and MD (eTable 6 in the Supplement).

### Primary Outcome

The mean (SD) number of mental health–related admissions in the follow-up period was 0.65 (2.43) among those with discontinuous care, 0.44 (1.54) for no primary care, and 0.51 (2.26) for continuous care. Mean (SD) duration of follow-up was 48.4 (25.3) weeks. In the adjusted model, adolescents with discontinuous primary care during transition had moderately higher rates of mental health–related admission in young adulthood compared with those with continuous care (aRR, 1.20; 95% CI, 1.10-1.30) (Table 3). Adolescents with no primary care during transition also had higher rates of mental health–related admissions (aRR, 1.30; 95% CI, 1.08-1.56). Compared with MD, having SZ alone and SZ and comorbid MD were associated with significantly higher rates of admission following transition, whereas ED was associated with lower rates (aRR, 0.77; 95% CI, 0.64-0.92).

**Table 3.** Adjusted Poisson Regression With aRR of Mental Health–Related Admission After Transition (Ages 19-26 Years) According to Pattern of Primary Care During the Transition Period for 8409 Participants

Variable	aRR (95% CI)^a^
Pattern of primary care during transition (ages 17-18 y)	
Discontinuous primary care	1.20 (1.10-1.30)
No primary care	1.30 (1.08-1.56)
Continuous primary care	1 [Reference]
Sex	
Male	1 [Reference]
Female	0.94 (0.87-1.03)
Rural	
Yes	0.90 (0.79-1.02)
No	1 [Reference]
Income quintile	
1 (Lowest)	1.26 (1.11-1.41)
2	1.17 (1.04-1.33)
3	0.97 (0.86-1.11)
4	1.06 (0.94-1.20)
5 (Highest)	1 [Reference]
Type of mental illness	
Schizophrenia^b^ alone	1.64 (1.45-1.84)
Schizophrenia^b^ and mood disorder	1.39 (1.22-1.58)
Eating disorder alone	0.77 (0.64-0.92)
Eating disorder and mood disorder	0.93 (0.79-1.08)
Mood disorder alone	1 [Reference]
Mental health–related admissions before transition (ages 12-16 y), No.	
0	1 [Reference]
1	1.26 (1.13-1.40)
2	1.42 (1.25-1.61)
≥3	2.06 (1.85-2.28)
Mental health–related admissions during transition (ages 17-18 y), No.	
0	1 [Reference]
1	2.46 (2.21-2.73)
2	2.48 (2.15-2.86)
≥3	5.49 (4.93-6.11)
Mental health–related visits by specialty during transition (ages 17-18 y)	
Psychiatrist (any)	3.12 (2.65-3.69)
Family physician and/or pediatrician	1.95 (1.62-2.34)
No mental health visits	1 [Reference]

Abbreviation: aRR, adjusted relative rate.

^a^ Adjusted for pattern of primary care during transition, sex, rurality, income quintile, type of mental illness, mental health–related admissions before and during transition, and mental health–related visits by specialty during transition.

^b^ Includes schizophrenia, delusional, and nonorganic psychotic disorder.

Our modeling approach revealed that the addition of mental health visits by specialty during transition had a strong confounding effect on the no primary care exposure (eTable 3 in the Supplement) as the aRR changed from 0.97 (95% CI, 0.81-1.16) to 1.30 (95% CI, 1.08-1.56). To understand this phenomenon, we examined the pattern of primary care continuity of mental health–related visits by physician specialty during transition and by the rate of mental health–related admissions following transition. Youths with no primary care practitioner during transition who also had no mental health visits following transition had more than double the rate of subsequent mental health–related admissions compared with youths with the same or different primary care practitioner during transition, explaining the confounding.

### Secondary Outcomes

Overall, the mean (SD) number of mental–health related emergency department visits in the follow-up period was 0.46 (1.06). Compared with having continuous primary care during transition, discontinuous care was associated with significantly higher rates of mental health–related emergency department visits in young adulthood (aRR, 1.16; 95% CI, 1.08-1.26) (Table 4). The overall number of deaths following transition was 69. This corresponds to a rate of 0.54 per 100 person-years of follow-up. Data on cause of death were available for 41 young adults, 54% of whom died of suicide, self-inflicted injuries, accidental poisonings, or transport accidents.

**Table 4.** Adjusted Poisson Regression With aRR of Mental Health–Related Emergency Department Visit After Transition (Ages 19-26 Years) According to Pattern of Primary Care During the Transition Period for 8409 Participants

Variable	aRR (95% CI)^a^
Pattern of primary care during transition (ages 17-18 y)	
Discontinuous primary care	1.16 (1.08-1.26)
No primary care	1.17 (0.98-1.39)
Continuous primary care	1 [Reference]
Sex	
Female	1.05 (0.97-1.14)
Male	1 [Reference]
Rural	
Yes	1.12 (1.00-1.25)
No	1 [Reference]
Income quintile	
1 (Lowest)	1.13 (1.01-1.26)
2	1.00 (0.89-1.12)
3	1.04 (0.93-1.17)
4	0.97 (0.87-1.08)
5 (Highest)	1 [Reference]
Type of mental illness	
Schizophrenia^b^ alone	1.04 (0.91-1.19)
Schizophrenia^b^ and mood disorder	0.89 (0.76-1.05)
Eating disorder alone	0.67 (0.58-0.78)
Eating disorder and mood disorder	1.04 (0.91-1.20)
Mood disorder alone	1 [Reference]
Mental health–related admissions before transition (ages 12-16 y), No.	
0	1 [Reference]
1	1.14 (1.04-1.25)
2	1.28 (1.14-1.44)
≥3	1.48 (1.34-1.65)
Mental health–related admissions during transition (ages 17-18 y), No.	
0	1 [Reference]
1	1.58 (1.43-1.75)
2	1.87 (1.63-2.15)
≥3	2.82 (2.51-3.18)
Mental health–related visits by specialty during transition (ages 17-18 y)	
Psychiatrist (any)	1.69 (1.51-1.90)
Family physician and/or pediatrician	1.47 (1.29-1.66)
No mental health visits	1 [Reference]

^a^ Adjusted for pattern of primary care during transition, sex, rurality, income quintile, type of mental illness, mental health–related admissions before and during transition, and mental health–related visits by specialty during transition.

^b^ Includes schizophrenia, delusional, and nonorganic psychotic disorder.

Subgroup analyses of youth with a family physician as their usual primary care practitioner at baseline (ages 12-16 years) revealed comparable estimates for the primary and secondary outcomes (eTable 4 and eTable 5 in the Supplement).

## Discussion

In this large population-based cohort study of transition-aged youth with severe mental illness in a universal health care system, we have demonstrated that both access to and continuity with primary care physicians during the period of transition to adult care was associated with improved outcomes in young adulthood. Although reports published by the American Academy of Pediatrics, American Academy of Family Physicians, and American College of Physicians have recommended primary care practitioners and medical specialists adopt a transition planning algorithm for all youth within a medical home specific to their clinical setting, the clinical reality and current emphasis in the transition literature focuses solely on specialist-to-specialist practitioner transition.^26^ Our study suggests that primary care involvement in transitional care may be important for moderating or improving long-term health outcomes for youth with mental illness. To our knowledge, this has been described only once previously in a recent Ontario study^30^ of youth with diabetes where a gap in diabetes care of more than 12 months and no primary care visits during transition were associated with an increased risk of ketoacidosis or death.

The lack of substantial evidence for the role of primary care in transition care^31^ is significant as many youths with mental health conditions are treated in primary care. Population-level data from Ontario indicate that most mental health–related outpatient physician care is provided by family physicians, with the highest visit rates among youth aged 18 to 21 years (31.8 visits per 100 population) and 22 to 24 years (39.9 visits per 100 population).^32^ The large role of family physicians in caring for youth’s mental health is consistent with our findings of care after transition to adult care.

While previous work in Ontario did not show significant changes in cost and use of services for youth with severe mental illness following transition to adult care, the follow-up window was only 2 years (age 20 years).^33^ Differences in pediatric care philosophies and approach may be, in part, contributing to our observations, but there may also be a lack of referrals to adult care. In a study of transition processes, although 80% of patients were deemed suitable, a third were not referred to adult mental health services.^14^ Another explanation for the decrease in psychiatry visits may be the natural history and prognosis of the various mental illnesses. Some youth in our study may simply have recovered from their illness (eg, MD and ED) and not require ongoing care. However, the findings of higher admission rates following transition in those with SZ suggest an important gap in care. For youth with ED, ambivalence about treatment and recovery is a core feature of their illness, even in the face of significant medical complications, and resistance to recovery is common.^34,35,36^ Therefore, less follow-up care may be a means of maintaining their eating disorder,^35,37^ so our data may not equate with recovery.

### Limitations

This study was limited to a population of transition-aged youth with the most severe presentation of mental illness. Our outpatient data are also limited to services billed by physicians and, thereby, do not capture care by others such as psychologists, social workers, nurses, and other therapists. In addition, we excluded patients whose primary care practitioner was in community health centers, as they are salaried and not fee-for-service; these centers provide care for more marginalized patients and this may limit generalizability of findings. Our data also cannot be generalized to youth who moved out of the province during the study period. Some youth, however, may have maintained Ontario residency while obtaining health services out-of-province as postsecondary students, which may have underestimated health care use after transition. It has been estimated that among the 60% of Canadians who attend postsecondary institutions, only approximately 10% attend out-of-province, and many still access health care in their own province.^38,39^ It is, therefore, unlikely that this had a substantial effect on our study results. Some telemedicine mental health services, used for youth in remote areas with limited access to psychiatry, are also not captured. Residual confounding from unknown or unmeasured covariates such as ethnic background, family composition, social supports, school-related factors, therapeutic approach, or individual level income may exist. The lack of primary care association may also be confounded by youth with poor overall functioning who are less capable of adhering to recommended treatment strategies and mental health appointments.

## Conclusions

Patterns of mental health care use for young adults with childhood-onset severe mental illness change during transition. Fragmented or no primary care during transition is associated with increased mental health admissions and emergency department visits after transition to adult care services. Timely and appropriate access to effective primary care during this period may help improve long-term outcomes and should be the focus of more research and policy making.

## Notes

Supplement. **eTable 1.** Databases Used to Derive Study Variables **eTable 2.** List of Data Sources and Codes Used to Define Key Variables **eTable 3.** Sequential Poisson Regression Models for Mental Health-Related Admissions After Transition (Age 19-26) According to Pattern of Primary Care During Transition **eTable 4.** Poisson Regression With Relative Rate (RR) of Mental Health-Related Admission After Transition (Age 19-26) Among Youth With a Family Physician as Their Usual Provider of Primary Care at Baseline, n=7,300 **eTable 5.** Poisson Regression With Relative Rate (RR) of Mental Health-Related Emergency Department Visit After Transition (Age 19-26) Among Youth With a Family Physician as Their Usual Provider of Primary Care at Baseline, n=7,300 **eTable 6.** Health Service Use Among Ontario Youth With Mental Illness Before (Age 12-16), During (Age 17-18), and After the Transition Period to Adult Care (Age 19-26), n=8,409
