# Supplementary material for: Association of Primary Care Continuity With Outcomes Following Transition to Adult Care for Adolescents With Severe Mental Illness
Source: JAMA Netw Open. 2019 Aug 2;2(8):e198415. doi: 10.1001/jamanetworkopen.2019.8415 (PMC6681550; doi:10.1001/jamanetworkopen.2019.8415)
Supplement: Supplement. — eTable 1. Databases Used to Derive Study Variables eTable 2. List of Data Sources and Codes Used to Define Key Variables eTable 3. Sequential Poisson Regression Models for Mental Health-Related Admissions After Transition (Age 19-26) According to Pattern of Primary Care During Transition eTable 4. Poisson Regression With Relative Rate (RR) of Mental Health-Related Admission After Transition (Age 19-26) Among Youth With a Family Physician as Their Usual Provider of Primary Care at Baseline, n=7,300 eTable 5. Poisson Regression With Relative Rate (RR) of Mental Health-Related Emergency Department Visit After Transition (Age 19-26) Among Youth With a Family Physician as Their Usual Provider of Primary Care at Baseline, n=7,300 eTable 6. Health Service Use Among Ontario Youth With Mental Illness Before (Age 12-16), During (Age 17-18), and After the Transition Period to Adult Care (Age 19-26), n=8,409 [file jamanetwopen-2-e198415-s001.pdf]

Supplementary Online Content

Toulany A, Stukel TA, Kurdyak P, Fu L, Guttmann A. Association of primary care continuity with outcomes following transition to adult care for adolescents with severe mental illness. *JAMA Netw Open*. 2019;2(8):e198415. doi:10.1001/jamanetworkopen.2019.8415

eTable 1. Databases Used to Derive Study Variables

eTable 2. List of Data Sources and Codes Used to Define Key Variables

eTable 3. Sequential Poisson Regression Models for Mental Health-Related Admissions After Transition (Age 19-26) According to Pattern of Primary Care During Transition

eTable 4. Poisson Regression With Relative Rate (RR) of Mental Health-Related Admission After Transition (Age 19-26) Among Youth With a Family Physician as Their Usual Provider of Primary Care at Baseline, n=7,300

eTable 5. Poisson Regression With Relative Rate (RR) of Mental Health-Related Emergency Department Visit After Transition (Age 19-26) Among Youth With a Family Physician as Their Usual Provider of Primary Care at Baseline, n=7,300

eTable 6. Health Service Use Among Ontario Youth With Mental Illness Before (Age 12-16), During (Age 17-18), and After the Transition Period to Adult Care (Age 19-26), n=8,409

This supplementary material has been provided by the authors to give readers additional information about their work.

© 2019 Toulany A et al. *JAMA Network Open.*

eTable 1: Databases used to derive study variables

Database	Abbreviation	Data Elements
Canadian Institute for Health Information Discharge Abstract Database	CIHI-DAD	Clinical data (diagnoses, procedures, physician); demographic data (patient gender, date of birth, postal code, county and residence code); administrative data (institution/hospital number, admission category, length of stay, disposition). Additional data held on mental health inpatients.
Ontario Mental Health Reporting System	OMHRS	Mental health clinical and administrative data on inpatient hospitalizations to designated adult mental health beds in psychiatric facilities from 68 participating hospitals in Ontario. Includes information about mental and physical health, social support and service use.
National Ambulatory Care Reporting System	NACRS	Data on patient visits to hospital and community based ambulatory care: day surgery, outpatient clinics and emergency departments.
Ontario Health Insurance Plan Claims Database	OHIP	Physician services paid within a fee-for-service system by the Ontario Ministry of Health and Long-Term Care. These services include physician consultations and assessments in acute care settings, office settings, and long-term care facilities; technical and professional components of diagnostic and therapeutic procedures; surgical procedures; and laboratory services.
Registered Persons Database	RPDB	Demographic information for all Ontarians with a valid health card. Variables obtained include date of birth, sex, and date of death (where applicable).
ICES Physician Database	IPDB	Physician specialty.
Office of the Registrar General – Deaths database	ORGD	Cause of death (available up until 2012).
Canadian Census Data		Neighbourhood income quintile and rural residence. Postal codes were linked to the Census to obtain mean neighbourhood income quintile, adjusted for both household and community size, for each dissemination area covering a population of 400–700.

© 2019 Toulany A et al. *JAMA Network Open.*

eTable 2: List of data sources and codes used to define key variables

Variable type	Data source(s)	Code(s)
Outcomes		
Mental health-related hospitalizations	CIHI-DAD, OMHRS	Any mental health code (ICD-10 F00-F99) as most responsible diagnosis was used in CIHI-DAD and any discharge diagnosis was used in OMHRS.
Mental health-related emergency department visits	NACRS	Any mental health code (ICD-10 F10-F99) in the main diagnostic field or a firm self-harm code (ICD-10 X60-X84) in any diagnostic field.
Death	RPDB, ORGD	Data on cause of death were obtained from the Office of the Registrar General – Deaths (ORGD) database up until 2012 and classified at the Institute for Clinical Evaluative Sciences using the International Classification of Diseases, 9th revision (ICD-9).
Exposure		
Pattern of primary care during transition	OHIP, IPDB	Baseline primary care provider [either GP/FP (specialty code 00) or paediatrician (specialty code 26)] was assigned at age 17.

© 2019 Toulany A et al. *JAMA Network Open.*

eTable 3: Sequential Poisson regression models for mental health-related admissions after transition (age 19-26) according to pattern of primary care during transition

Step 1: Addition of sex			
Variable		Unadjusted RR (95% CI)	Adjusted RR (95% CI)
Pattern of primary care (PC) during transition			
	Different PC provider	1.29 (1.17, 1.41)	1.26 (1.15, 1.39)
	No PC provider	0.93 (0.76, 1.14)	0.85 (0.70, 1.04)
	Same PC provider	Reference	Reference
Sex			
	Female	0.73 (0.67, 0.80)	0.73 (0.67, 0.80)
	Male	Reference	Reference
Step 2: Addition of rural residence and income quintile			
Variable		Unadjusted RR (95% CI)	Adjusted RR (95% CI)
Pattern of primary care (PC) during transition			
	Different PC provider	1.29 (1.17, 1.41)	1.25 (1.14, 1.37)
	No PC provider	0.93 (0.76, 1.14)	0.83 (0.68, 1.02)
	Same PC provider	Reference	Reference
Sex			
	Female	0.73 (0.67, 0.80)	0.74 (0.68, 0.81)
	Male	Reference	Reference
Rural			
	Yes	0.79 (0.68, 0.92)	0.78 (0.67, 0.91)
	No	Reference	Reference
Income Quintile			
	1 (lowest)	1.54 (1.34, 1.76)	1.54 (1.35, 1.77)
	2	1.27 (1.10, 1.47)	1.26 (1.10, 1.46)
	3	1.10 (0.95, 1.27)	1.10 (0.95, 1.27)
	4	1.18 (1.03, 1.37)	1.19 (1.03, 1.37)
	5 (highest)	Reference	Reference

© 2019 Toulany A et al. *JAMA Network Open.*

Step 3: Addition of type of mental illness			
Variable		Unadjusted RR (95% CI)	Adjusted RR (95% CI)
Pattern of primary care (PC) during transition			
	Different PC provider	1.29 (1.17, 1.41)	1.22 (1.11, 1.33)
	No PC provider	0.93 (0.76, 1.14)	0.80 (0.66, 0.98)
	Same PC provider	Reference	Reference
Sex			
	Female	0.73 (0.67, 0.80)	0.93 (0.85, 1.03)
	Male	Reference	Reference
Rural			
	Yes	0.79 (0.68, 0.92)	0.77 (0.67, 0.90)
	No	Reference	Reference
Income Quintile			
	1 (lowest)	1.54 (1.34, 1.76)	1.34 (1.18, 1.54)
	2	1.27 (1.10, 1.47)	1.17 (1.02, 1.35)
	3	1.10 (0.95, 1.27)	1.02 (0.88, 1.17)
	4	1.18 (1.03, 1.37)	1.12 (0.97, 1.28)
	5 (highest)	Reference	Reference
Type of mental illness			
	SZ alone	2.88 (2.55, 3.26)	2.75 (2.41, 3.12)
	SZ & MAD	3.56 (3.10, 4.09)	3.45 (3.01, 3.97)
	ED alone	0.53 (0.43, 0.65)	0.55 (0.45, 0.67)
	ED & MAD	1.35 (1.14, 1.60)	1.39 (1.17, 1.65)
	MAD alone	Reference	Reference

Step 4: Addition of mental health admissions before transition (age 12-16)			
Variable		Unadjusted RR (95% CI)	Adjusted RR (95% CI)
Pattern of primary care (PC) during transition			
	Different PC provider	1.29 (1.17, 1.41)	1.14 (1.05, 1.25)
	No PC provider	0.93 (0.76, 1.14)	0.83 (0.68, 1.00)
	Same PC provider	Reference	Reference
Sex			
	Female	0.73 (0.67, 0.80)	0.92 (0.84, 1.01)
	Male	Reference	Reference
Rural			
	Yes	0.79 (0.68, 0.92)	0.79 (0.69, 0.91)
	No	Reference	Reference
Income Quintile			

© 2019 Toulany A et al. *JAMA Network Open.*

	1 (lowest)	1.54 (1.34, 1.76)	1.28 (1.13, 1.46)
	2	1.27 (1.10, 1.47)	1.18 (1.03, 1.35)
	3	1.10 (0.95, 1.27)	1.00 (0.87, 1.15)
	4	1.18 (1.03, 1.37)	1.11 (0.97, 1.27)
	5 (highest)	Reference	Reference
Type of mental illness			
	SZ alone	2.88 (2.55, 3.26)	2.44 (2.15, 2.77)
	SZ & MAD	3.56 (3.10, 4.09)	2.04 (1.78, 2.35)
	ED alone	0.53 (0.43, 0.65)	0.64 (0.53, 0.78)
	ED & MAD	1.35 (1.14, 1.60)	1.04 (0.88, 1.23)
	MAD alone	Reference	Reference
Mental health admissions before transition (12-16 y)			
	1	1.57 (1.40, 1.76)	1.46 (1.31, 1.64)
	2	2.30 (2.01, 2.64)	2.02 (1.77, 2.31)
	≥3	4.98 (4.48, 5.53)	4.03 (3.61, 4.49)
	None	Reference	Reference

© 2019 Toulany A et al. *JAMA Network Open.*

Step 5: Addition of mental health admission during transition (age 17-18)			
Variable		Unadjusted RR (95% CI)	Adjusted RR (95% CI)
Pattern of primary care (PC) during transition			
	Different PC provider	1.29 (1.17, 1.41)	1.16 (1.07, 1.26)
	No PC provider	0.93 (0.76, 1.14)	0.97 (0.81, 1.16)
	Same PC provider	Reference	Reference
Sex			
	Female	0.73 (0.67, 0.80)	0.93 (0.86, 1.01)
	Male	Reference	Reference
Rural			
	Yes	0.79 (0.68, 0.92)	0.84 (0.73, 0.95)
	No	Reference	Reference
Income Quintile			
	1 (lowest)	1.54 (1.34, 1.76)	1.17 (1.04, 1.33)
	2	1.27 (1.10, 1.47)	1.14 (1.00, 1.29)
	3	1.10 (0.95, 1.27)	0.94 (0.83, 1.07)
	4	1.18 (1.03, 1.37)	1.04 (0.92, 1.18)
	5 (highest)	Reference	Reference
Type of mental illness			
	SZ alone	2.88 (2.55, 3.26)	1.76 (1.56, 1.98)
	SZ & MAD	3.56 (3.10, 4.09)	1.46 (1.28, 1.67)
	ED alone	0.53 (0.43, 0.65)	0.74 (0.62, 0.89)
	ED & MAD	1.35 (1.14, 1.60)	0.97 (0.83, 1.13)
	MAD alone	Reference	Reference
Mental health admissions before transition (12-16 y)			
	1	1.57 (1.40, 1.76)	1.30 (1.17, 1.45)
	2	2.30 (2.01, 2.64)	1.54 (1.36, 1.75)
	≥3	4.98 (4.48, 5.53)	2.21 (1.98, 2.46)
	None	Reference	Reference
Mental health admissions during transition (17-18 y)			
	1	3.85 (3.47, 4.27)	3.23 (2.91, 3.57)
	2	4.37 (3.80, 5.03)	3.21 (2.78, 3.69)
	≥3	11.73 (10.66, 12.91)	7.37 (6.63, 8.18)
	None	Reference	Reference

© 2019 Toulany A et al. *JAMA Network Open.*

Step 6: Addition of mental health visits by specialty during transition (age 17-19)			
Variable		Unadjusted RR (95% CI)	Adjusted^ RR (95% CI)
Pattern of primary care (PC) during transition			
	Different PC provider	1.29 (1.17, 1.41)	1.20 (1.10, 1.30)
	No PC provider	0.93 (0.76, 1.14)	1.30 (1.08, 1.56)
	Same PC provider	Reference	Reference
Sex			
	Female	0.73 (0.67, 0.80)	0.94 (0.87, 1.03)
	Male	Reference	Reference
Rural			
	Yes	0.79 (0.68, 0.92)	0.90 (0.79, 1.02)
	No	Reference	Reference
Income Quintile			
	1 (lowest)	1.54 (1.34, 1.76)	1.26 (1.11, 1.41)
	2	1.27 (1.10, 1.47)	1.17 (1.04, 1.33)
	3	1.10 (0.95, 1.27)	0.97 (0.86, 1.11)
	4	1.18 (1.03, 1.37)	1.06 (0.94, 1.20)
	5 (highest)	Reference	Reference
Type of mental illness			
	SZ* alone	2.88 (2.55, 3.26)	1.64 (1.45, 1.84)
	SZ* & MAD	3.56 (3.10, 4.09)	1.39 (1.22, 1.58)
	ED alone	0.53 (0.43, 0.65)	0.77 (0.64, 0.92)
	ED & MAD	1.35 (1.14, 1.60)	0.93 (0.79, 1.08)
	MAD alone	Reference	Reference
Mental health admissions before transition (12-16 y)			
	1	1.57 (1.40, 1.76)	1.26 (1.13, 1.40)
	2	2.30 (2.01, 2.64)	1.42 (1.25, 1.61)
	≥3	4.98 (4.48, 5.53)	2.06 (1.85, 2.28)
	None	Reference	Reference
Mental health admissions during transition (17-18 y)			
	1	3.85 (3.47, 4.27)	2.46 (2.21, 2.73)
	2	4.37 (3.80, 5.03)	2.48 (2.15, 2.86)
	≥3	11.73 (10.66, 12.91)	5.49 (4.93, 6.11)
	None	Reference	Reference
Mental health visits by specialty during transition (17-18 y)			
	Psychiatrist (any)	6.48 (5.46, 7.68)	3.12 (2.65, 3.69)
	General Practitioner and/or Paediatrician	2.07 (1.69, 2.52)	1.95 (1.62, 2.34)
	No mental health visits	Reference	Reference

© 2019 Toulany A et al. *JAMA Network Open.*

abbreviations: SZ: schizophrenia/delusional/non-organic psychotic disorder, ED: eating disorder, MAD: mood/affective disorders

*Includes schizophrenia, delusional, non-organic psychotic disorder

^Adjusted for pattern of primary care during transition, sex, rurality, income quintile, type of mental illness, mental health admissions before and during transition, and mental health visits by specialty during transition

© 2019 Toulany A et al. *JAMA Network Open.*

eTable 4: Poisson regression with relative rate (RR) of mental health-related admission after transition (age 19-26) among youth with a family physician as their usual provider of primary care at baseline, n=7,300

Variable		Unadjusted RR (95% CI)	Adjusted^ RR (95% CI)
Pattern of primary care (PC) during transition			
	Different PC provider	1.47 (1.33, 1.64)	1.33 (1.21, 1.46)
	No PC provider	0.91 (0.71, 1.16)	1.28 (1.03, 1.60)
	Same PC provider	Reference	Reference
Sex			
	Female	0.75 (0.68, 0.82)	0.97 (0.88, 1.06)
	Male	Reference	Reference
Rural			
	Yes	0.71 (0.60, 0.84)	0.78 (0.68, 0.90)
	No	Reference	Reference
Income Quintile			
	1 (lowest)	1.57 (1.36, 1.82)	1.27 (1.12, 1.44)
	2	1.19 (1.02, 1.39)	1.09 (0.96, 1.25)
	3	1.10 (0.94, 1.28)	0.96 (0.84, 1.10)
	4	1.19 (1.02, 1.39)	1.06 (0.93, 1.21)
	5 (highest)	Reference	Reference
Type of mental illness			
	SZ* alone	3.09 (2.71, 3.53)	1.73 (1.53, 1.97)
	SZ* & MAD	3.70 (3.20, 4.28)	1.37 (1.19, 1.57)
	ED alone	0.56 (0.45, 0.70)	0.83 (0.69, 1.01)
	ED & MAD	1.44 (1.20, 1.72)	0.96 (0.82, 1.13)
	MAD alone	Reference	Reference
Mental health admissions before transition (12-16 y)			
	1	1.54 (1.36, 1.74)	1.23 (1.10, 1.38)
	2	2.20 (1.90, 2.56)	1.39 (1.21, 1.60)
	≥3	5.32 (4.76, 5.95)	2.22 (1.99, 2.48)
	None	Reference	Reference
Mental health admissions during transition (17-18 y)			
	1	3.86 (3.45, 4.31)	2.42 (2.16, 2.72)
	2	4.10 (3.52, 4.78)	2.28 (1.96, 2.66)
	≥3	11.72 (10.57, 13.00)	5.23 (4.66, 5.87)
	None	Reference	Reference
Mental health visits by specialty during transition (17-18 y)			
	Psychiatrist (any)	6.88 (5.68, 8.33)	3.19 (2.65, 3.83)
	General Practitioner and/or Paediatrician	2.11 (1.69, 2.62)	1.99 (1.63, 2.43)
	No mental health visits	Reference	Reference

© 2019 Toulany A et al. *JAMA Network Open.*

abbreviations: SZ: schizophrenia/delusional/non-organic psychotic disorder, ED: eating disorder, MAD: mood/affective disorders

*Includes schizophrenia, delusional, non-organic psychotic disorder

^Adjusted for pattern of primary care during transition, sex, rurality, income quintile, type of mental illness, mental health admissions before and during transition, and mental health visits by specialty during transition

© 2019 Toulany A et al. *JAMA Network Open.*

eTable 5: Poisson regression with relative rate (RR) of mental health-related emergency department visit after transition (age 19-26) among youth with a family physician as their usual provider of primary care at baseline, n=7,300

Variable		Unadjusted RR (95% CI)	Adjusted^RR (95% CI)
Pattern of primary care (PC) during transition			
	Different PC provider	1.25 (1.14, 1.37)	1.23 (1.13, 1.35)
	No PC provider	0.67 (0.53, 0.85)	0.86 (0.69, 1.09)
	Same PC provider	Reference	Reference
Sex			
	Female	0.97 (0.89, 1.06)	1.05 (0.96, 1.14)
	Male	Reference	Reference
Rural			
	Yes	1.04 (0.92, 1.18)	1.10 (0.98, 1.24)
	No	Reference	Reference
Income Quintile			
	1 (lowest)	1.21 (1.07, 1.37)	1.12 (0.99, 1.26)
	2	1.02 (0.89, 1.16)	0.98 (0.86, 1.11)
	3	1.07 (0.95, 1.21)	1.03 (0.91, 1.16)
	4	1.02 (0.90, 1.16)	0.96 (0.85, 1.09)
	5 (highest)	Reference	Reference
Type of mental illness			
	SZ* alone	1.41 (1.21, 1.63)	1.05 (0.90, 1.22)
	SZ* & MAD	1.60 (1.35, 1.90)	0.95 (0.80, 1.12)
	ED alone	0.62 (0.53, 0.74)	0.74 (0.63, 0.86)
	ED & MAD	1.39 (1.20, 1.61)	1.11 (0.96, 1.28)
	MAD alone	Reference	Reference
Mental health admissions before transition (12-16 y)			
	1	1.30 (1.17, 1.43)	1.17 (1.06, 1.28)
	2	1.65 (1.46, 1.88)	1.30 (1.15, 1.47)
	≥3	2.38 (2.14, 2.64)	1.50 (1.34, 1.68)
	None	Reference	Reference
Mental health admissions during transition (17-18 y)			
	1	1.95 (1.75, 2.17)	1.56 (1.40, 1.75)
	2	2.48 (2.15, 2.86)	1.91 (1.65, 2.21)
	≥3	4.00 (3.57, 4.48)	2.82 (2.49, 3.21)
	None	Reference	Reference
Mental health visits by specialty during transition (17-18 y)			
	Psychiatrist (any)	2.45 (2.18, 2.76)	1.68 (1.48, 1.90)
	General Practitioner and/or Paediatrician	1.54 (1.35, 1.76)	1.44 (1.26, 1.64)
	No mental health visits	Reference	Reference

© 2019 Toulany A et al. *JAMA Network Open.*

abbreviations: SZ: schizophrenia/delusional/non-organic psychotic disorder, ED: eating disorder, MAD: mood/affective disorders

*Includes schizophrenia, delusional, non-organic psychotic disorder

^Adjusted for pattern of primary care during transition, sex, rurality, income quintile, type of mental illness, mental health admissions before and during transition, and mental health visits by specialty during transition

© 2019 Toulany A et al. *JAMA Network Open.*

eTable 6: Health service use among Ontario youth with mental illness before (age 12-16), during (age 17-18), and after the transition period to adult care (age 19-26), n=8,409

	Schizophrenia° n=804			Eating Disorders n=1,403			Mood Disorders n=6,202		
Variable	12-16 y	17-18 y	19-26 y	12-16 y	17-18 y	19-26 y	12-16 y	17-18 y	19-26 y
Overall health service use, mean annualized rates (SD)									
Primary care visits	3.35 (2.75)	3.58 (3.69)	3.69 (4.00)	6.18 (6.31)	5.72 (7.46)	4.58 (6.18)	3.98 (3.11)	4.32 (4.25)	4.01 (3.91)
UPC visits	1.59 (1.77)	1.46 (2.31)	1.06 (1.83)	2.17 (2.23)	2.16 (2.97)	1.31 (2.11)	1.97 (1.91)	1.90 (2.51)	1.22 (2.09)
Mental health-related service use, mean annualized rates (SD)									
MH FP visits	0.81 (1.16)	1.19 (1.96)	1.62 (3.98)	1.40 (2.36)	1.59 (3.08)	1.57 (3.44)	0.96 (1.22)	1.22 (3.15)	1.74 (5.11)
MH paediatrician visits	0.33 (0.65)	0.12 (0.46)	0.02 (0.19)	2.46 (6.10)	1.47 (6.90)	0.02 (0.24)	0.33 (0.85)	0.15 (0.84)	0.02 (0.32)
MH psychiatrist visits	2.99 (4.77)	4.74 (6.57)	3.11 (4.49)	2.97 (5.36)	3.17 (8.49)	1.53 (4.05)	1.83 (3.49)	2.13 (4.98	1.31 (3.59)
MH admissions	0.32 (0.39)	0.51 (0.94)	0.37 (0.76)	0.19 (0.33)	0.18 (0.64)	0.11 (0.41)	0.17 (0.29)	0.17 (0.51)	0.12 (0.65)
MH ED visits	0.11 (0.18)	0.19 (0.34)	0.18 (0.39)	0.09 (0.17)	0.10 (0.27)	0.11 (0.31)	0.13 (0.19)	0.13 (0.32)	0.12 (0.29)

© 2019 Toulany A et al. *JAMA Network Open.*
